# Australian medical students' perceptions of professionalism and ethics in medical television programs

**DOI:** 10.1186/1472-6920-11-50

**Published:** 2011-07-29

**Authors:** Roslyn Weaver, Ian Wilson

**Affiliations:** 1 Family and Community Health Research Group, University of Western Sydney, Narellan Road Campbelltown NSW 2560, Australia; 2 Medical Education Unit, School of Medicine, University of Western Sydney, Narellan Road Campbelltown NSW 2560, Australia

## Abstract

**Background:**

Medical television programs offer students fictional representations of their chosen career. This study aimed to discover undergraduate medical students' viewing of medical television programs and students' perceptions of professionalism, ethics, realism and role models in the programs. The purpose was to consider implications for teaching strategies.

**Methods:**

A medical television survey was administered to 386 undergraduate medical students across Years 1 to 4 at a university in New South Wales, Australia. The survey collected data on demographics, year of course, viewing of medical television programs, perception of programs' realism, depiction of ethics, professionalism and role models.

**Results:**

The shows watched by most students were *House, Scrubs*, and *Grey's Anatomy*, and students nominated watching 30 different medical programs in total. There was no statistical association between year of enrolment and perceptions of accuracy. The majority of students reported that friends or family members had asked them for their opinion on an ethical or medical issue presented on a program, and that they discussed ethical and medical matters with their friends. Students had high recall of ethical topics portrayed on the shows, and most believed that medical programs generally portrayed ideals of professionalism well.

**Conclusions:**

Medical programs offer considerable currency and relevance with students and may be useful in teaching strategies that engage students in ethical lessons about practising medicine.

## Background

Popular images of particular careers can have an impact on students' decisions to enter and remain in a profession. Students in professional courses can often develop their sense of professional identity early in their studies [1]. Anecdotally, images of medical doctors in popular culture may have some influence on students' professional identity, particularly in television programs. Research suggests that medical televisual texts can affect students' expectations of the medical profession, whereby fictional doctors in programs such as *ER *may influence medical students' perceptions and beliefs in a similar way to the real doctors they encounter [2]. These images can potentially help to shape students' expectations of medical practice, ethics and professional identity even before they make their decision to enrol in a medical degree.

Images of the medical world have been researched from a variety of perspectives, such as Turow's [3] work on television doctors. While multiple media can affect students' perceptions of their profession, television is by far the most dominant medium because of its wide accessibility. Yet the evidence is mixed regarding the impact on students who watch these medical programs and the pedagogical value of popular culture texts in medical education. Some researchers suggest the programs can positively influence students and are useful teaching strategies [4,5]. Previous studies suggest that medical television dramas can be used to teach improved communication skills [6] and to promote discussions of ethics [7-9]. Meanwhile, other research raises concerns about negative aspects of television programs [10-14], suggesting they may be irrelevant and unrealistic at best and harmful and dangerous at worst.

In a study of medical and nursing students at an American university, Czarny et al. [4] showed that the majority of students watched medical television programs (81% of medical students and 84% of nursing students), with the most watched shows including *House *and *Grey's Anatomy*. In their attitudes to the ethical dilemmas portrayed on these programs, pre-clinical medical students were more likely to believe such dilemmas were handled adequately than nursing or post-clinical medical students [4]. As Czarny et al. [4] note, further research in this area is needed to assess the influences on medical students in their studies and attitudes to their profession. However, to date there has been little work done in the Australian context as a comparative measure. The aim of our study, therefore, was to explore undergraduate medical students' perceptions of medical television programs in the Australian setting. This paper reports the results of a survey of medical students on their viewing and perceptions of medical television shows.

## Methods

This study took place at an undergraduate medical school at a large university in New South Wales, Australia. Students received an email or announcement advertising the project. All students across Years 1 to 4 were invited to join the project and participants completed surveys after lectures and tutorials at the start of Semester 1 between January and March in 2010.

We used an adapted form of Czarny et al.'s [4] medical television drama survey. Unlike the group surveyed by Czarny et al. [4], the participants in our study were all undergraduate medical students enrolled in a five-year course; at the time of data collection, Year 4 was the first cohort of this school and therefore there was no Year 5. We did not survey nursing students in this study. The survey adaptations were limited to the addition of Australian television programs (*All Saints*), more open-ended questions relating to role models, and more prompts (giving examples of other shows). Several questions were rephrased in minor ways, such as changing "Do you watch television medical dramas" to "Do you watch medical television shows", or "Don't ever watch" to "Never watch". The survey asked for demographic information (age, gender, year of enrolment), television viewing habits (which medical television programs they watch and how frequently), and attitudes to television programs and fictional role models (perceptions of realism, which characters they aspire to be like the most and the least, and why, and other role models outside television). We pilot tested the revised survey on eight volunteers from medicine and nursing staff and postgraduate students. Based on this feedback, we reformatted the survey to improve clarity. This paper reports the statistical results of this survey, not the qualitative results.

### Statistical analysis

A research assistant entered the survey data into SPSS. The data analysis process involved comparison of groups using *Χ*^*2*^. Significance was set at α = 0.05. All analyses were conducted using SPSS 18.

### Ethical approval

The university human ethics research committee granted approval to conduct this study. The privacy of all participants was maintained at all times. Surveys were stored in a locked filing cabinet and the data were stored on a password-protected computer.

## Results

A total of 386 students completed the survey, representing 85.2% of the 453 students enrolled in the medical school at the time of collection. This represented 95.4% (n = 124) of the 130 students enrolled in first year, 73.8% (n = 96) of 130 students in second year, 78.3% (n = 83) of 106 students in third year, and 94.3% (n = 82) of 87 students in fourth year. One student did not report year of course. Student demographics are detailed in Table 1.

**Table 1:** Student demographics

Characteristic	
Age Mean Years	20.2

Sex (Male/Female)	172 (44.6%)/214 (55.4%)

Country of birth (Australia/Overseas)	207 (53.6%)/179 (46.4%)

Language spoken at home (English/Other/Both)	211 (54.7%)/54 (14.0%)/121 (31.3%)

Part-time employment during semester (Yes/No)	172 (44.6%)/214 (55.4%)

Watching television was very common with 99.5% of respondents watching television in the past year. The most commonly watched categories were films (98.7%), comedies (97.1%), news (96.2%) and medical shows (93.7%).

### Medical shows students watch

Students were asked to indicate the frequency with which they watched programs using the scale: Never, Only ever once or twice, I used to but not anymore, Occasionally, Less than once a week, Once a week or more. The main programs we included were: *House, Grey's Anatomy, Scrubs, ER, All Saints*, and *Private Practice*. *House *and *Scrubs *were the most frequently watched shows, while *Scrubs *was the most currently watched show (Table 2). For most medical shows, respondents reported watching the show primarily by themselves. *Scrubs *had a higher proportion of students (15.8%) watching it with other medical students.

**Table 2:** Television shows watched

Program	Watched at some stage*	Currently watch^**^	Watch mostly alone***	Watch mostly with medical friends***	Watch mostly with others***
*House*	93.7%	49.7%	53.4%	7.3%	39.3%

*Scrubs*	90.8%	65.3%	55.9%	15.8%	28.3%

*Grey's Anatomy*	66.4%	37.6%	55.0%	6.9%	38.1%

*ER*	40.3%	11.6%	53.7%	3.7%	42.6%

*Private Practice*	37.2%	13.8%	59.8%	5.5%	34.7%

*All Saints*	29.9%	7.4%	48.1%	1.0%	50.9%

* Watched at some stage = 'only ever once or twice', 'I used to, but not anymore'

** Currently watch = 'occasionally', 'less than once a week' and 'more than once a week'

*** Percentage of students who have watched the show

In addition to these six programs, students also nominated other medical television programs they had watched, and these included 24 drama, comedy and reality-documentary programs, with the highest ranked shows *RPA *(17.8%), *Nip/Tuck *(11.2%), M*A*S*H (2.2%), *Medical Emergency *(2.1%), *Nurse Jackie *(1.8%), *Mercy *(1.6%), *ICU: A Matter of Life and Death *(1.4%), *Three Rivers *(1.3%), and *Royal Pains *(1.1%). All others were under one percent.

Female students were more likely than male students to watch *Grey's Anatomy *(Females 48.2%, Males 23.7%, Χ^2 ^= 22.1, df = 1, p < 0.001) and *Private Practice *(Females 19.4%, Males 6.6%, Χ^2 ^= 11.7, df = 1, p = 0.001). There was no relationship between medical program watched and year of course.

### Role models and ideals of professionalism

Students were asked which characters on *Grey's Anatomy *and *House *they wished most and least to be like in their professional careers. These programs were chosen because they were the two major medical programs at the time. For *Grey's Anatomy*, 29.6% of students completing this question chose "none" as their answer; of the remaining students who nominated a character they most wished to be like, the two most popular answers were "Derek Shepherd" and "Miranda Bailey" (each 25.1%). In *House*, 13.7% answered "none"; of the remaining students, the favoured characters were "Cameron" (27.5%), "Wilson" (26.4%), and the lead character "House" (26.0%). When choosing characters students least wanted to be like, the most common responses were "Cristina Yang" (27.6%) and "Meredith Grey" (27.1%) in *Grey's Anatomy *and "House" (42.3%) in *House*.

The survey also collected students' opinions about which ideals of professionalism they remembered seeing in medical television programs generally and whether these ideals were depicted positively or negatively (Table 3). Between 78.2% and 86.0% of students recalled and rated each topic; of these, the majority rated the shows positively: leadership (83.4%), caring and compassion (83.1%), responsibility (82.2%), altruism/honesty/integrity (79.1%), respect (75.7%), and accountability (68.0%).

**Table 3:** Professional ideals represented in medical shows

Professional ideal	% students noted issue	Rated positively (% of students noting issue)	Rated negatively (% of students noting issue)
Caring and compassion	86.0%	83.1%	16.9%

Altruism, honesty, integrity	83.2%	79.1%	20.9%

Respect	82.1%	75.7%	24.3%

Responsibility	81.6%	82.2%	17.8%

Accountability	79.3%	68.0%	32.0%

Leadership	78.2%	83.4%	16.6%

### Ethical issues on television shows and perceptions of accuracy

Of the ethical issues presented on medical television shows, the most frequently recalled were medical mistakes (96.6%), death and dying (94.6%), professional misconduct (92%) and quality or value of life or personhood (91.1%). Students who recalled seeing particular ethical issues reported mixed assessments of accuracy (see Table 4). Issues that were most poorly portrayed (rated as Poor or Below Average) were human experimentation, body enhancement, and education for health professionals. Issues that were well portrayed (rated as OK or Best) were death and dying, and quality or value of life or personhood.

**Table 4:** Ethical issues represented in medical shows

Ethical issue	% students noted issue	Well presented* (% of students noting issue)	Poorly presented** (% of students noting issue)
Medical mistakes	96.6%	69.2%	30.8%

Death & dying	94.6%	77.5%	22.5%

Professional misconduct	92.0%	42.0%	58.0%

Quality or value of life	91.1%	73.8%	26.2%

Confidentiality	89.7%	43.3%	56.7%

Truth disclosure	88.8%	47.7%	52.3%

Informed consent	87.7%	46.1%	53.9%

Infectious disease	85.4%	55.5%	44.5%

Access & equity	80.7%	43.8%	56.2%

Education for health care professionals	78.9%	34.4%	65.6%

Rationing care	77.4%	67.8%	32.2%

Artificial and transplanted organs/tissues	77.1%	45.0%	55.0%

Enhancement (plastic surgery etc)	75.0%	35.3%	64.7%

Human experimentation	63.4%	29.1%	70.9%

* Well presented = 'OK', 'Best'

** Poorly presented = 'Poor', 'Below average'

A majority of students (55.9%) reported that their friends or family members asked them for their opinion on an ethical or medical issue they viewed on a medical program. Over half the students (60.1%) reported that they discussed ethical and medical issues from the medical programs with their friends. When asked to rank different sources informing them about bioethical issues, students predominantly relied on the medical school (72.9% ranking 1-3) and family (51.8% ranking 1-3) (see Table 5). TV medical dramas rated among the lowest of sources (8.8% ranking 1-3).

**Table 5:** Sources of bioethical advice for students

Source of information	Percent of students ranking 1 - 3 as source of advice
Medical school	72.9%

Family	51.8%

Scholarly journals	40.0%

Friends	38.6%

Newspapers	32.6%

Clergy/religious values	28.7%

TV News	22.4%

TV Medical Dramas	8.8%

Popular magazines	6.3%

Other*	11.1%

* Each less than 1% of students

Students were asked to rate the accuracy with which programs depicted ethical and medical issues that arise in practice (using a scale from 0 Not at all accurate to 5 Very accurate). Of the students who had seen the program, the proportion of students rating accuracy as 3-5 ranged from 35.0% for *Grey's Anatomy*, 35.6% for *Private Practice *and 47.6% for *House *to 52.8% for *All Saints*, 61.7% for *Scrubs *and 66.7% for *ER*. There was no statistical association between year of course and perception of accuracy.

## Discussion

The results of this study show that medical students are very high consumers of medical television programs, which confirms the findings of Czarny et al. [4] Female students were more likely than males to watch *Grey's Anatomy *(and its spin off, *Private Practice*), as in Czarny et al.'s [4] study, which perhaps reflects role modelling in that students may be more likely to watch a show featuring a protagonist of the same gender. Another similarity between our research and Czarny et al.'s [4] study was a high recall of ethical issues in both samples, although our sample showed a higher proportion of students watching medical programs and recalling ethical issues. The popularity of *Scrubs*, one of the most frequently watched of any medical program, and certainly the most popular of all shows to watch with fellow medical students, confirms both anecdotal evidence from the students of their enthusiasm for the show, and the qualitative results from this study, which are not reported here.

Our results showed no statistical association between year of enrolment and perception of accuracy. This finding differs to the American study, which noted that students with clinical experience tended to rate the shows more negatively (in both medicine and nursing). Yet this was not the case in our research. From this we might conclude that these students begin their studies from a more critical perspective about the realism of medical television programs, or, conversely, that medical television programs do not greatly influence their beliefs and expectations about the medical profession, or not in ways that our study could measure. Indeed, students gave a very low ranking to medical television shows as sources of learning about ethical issues, which implies that they do not believe the television programs influence them. However, this finding is at odds with the students' high consumption of these shows, recall of ethical issues portrayed, and beliefs about the professionalism on the shows. Indeed, the majority of students believed that all ideals of professionalism were depicted positively in medical programs. This finding is concerning given that previous research points out some of the negative aspects of these television shows, such as high levels of unprofessional behaviour [15], medical paternalism in *House *[10], stereotypes of gender in *Grey's Anatomy *[11], and the absence of nursing staff [12,16]. Given that ethical and legal principles are regularly abandoned in favour of drama in these programs, it is surprising that the majority of students felt that overall medical shows portrayed professionalism well. It may be that students do not realise the extent to which they are influenced by medical programs, even in subtle ways.

This research has implications for the educational setting. Anecdotally, teaching staff can be anxious about the impact of how the fictional world of medicine intrudes on and influences the real one. The high number of students who could remember ethical issues from the medical programs confirms the suggestion that there may be benefit in using the shows in tutorials or lectures as case studies or examples for students in medical education. Goodman [17] notes that television programs may be worthwhile tutorial teaching tools to assist in medical students' learning. As Spike [7] points out, television programs adhere to the classic Hollywood paradigm of a morality tale and may therefore be valuable aids to teaching students key lessons about ethics and professionalism, and in some ways can fill a gap where medical education does not adequately cover issues of ethics and professionalism in curricula. Our results show that a majority of students will discuss the medical and ethical issues raised in television programs with family and friends and each other anyway. If medical educators are willing to consider televisual texts as pedagogical tools, such as using specific scenes to illustrate particular lessons or to provoke debate, this may assist students to develop the analytical tools to not simply discuss the programs with others but to critically engage with the programs. There can be value in adopting a more inclusive approach to popular culture in medical education, to encourage critical thinking about ethical issues in medical practice.

Our research confirms, however, that there are negative features of medical television programs in the context of realism. In our study, students rated nine of the fourteen named ethical and medical issues as poorly represented in the shows, which reinforces that there are many important issues treated inappropriately on television. Yet the fact that students rated the realism negatively shows that they are able to engage critically with the programs to some extent. This is an important point. Students do not automatically acquire the skills to critically analyse texts such as television shows, and there is the potential for students and others to passively consume these programs. Although some researchers highlight the advantages of engaging with students via a medium they understand and enjoy, other commentators are dismissive of the benefits of using such a tool. For instance, Trachtman [13] questions medical television programs as teaching tools because of the "intrinsic flaws" in their presentation of ethical dramas (p.9). Trachtman [13] rightly points to the competing demands of realism and televisual narrative structure that can compromise medical authenticity. Not just ethics but practical skills can be a problem of realism in these programs. Deficient resuscitation skills have been linked to students absorbing inaccurate techniques from television programs such as *ER *[14]. Our results show that students rated *ER *highly for its perceived realism, which is perhaps due to its age - which may be likely to bestow more credibility given it was a long-running program - and its focus on a team of doctors working on cases, rather than one or two dominant characters and medical cases as on more recent programs. Whatever the flaws of realism in these programs, however, they do not negate the possible value of using televisual texts as point of departure exemplars for medical students. Indeed, it may be precisely because of their flawed realism that they are vital to use in discussions with students to lead to an understanding of best practice.

One of the strengths of this study is in its attention to popular images of medicine, an area that is often overlooked in research about medical practice, policy, and education. It is difficult to measure the influence of popular culture on medical students and the general public, yet this study demonstrates that students are not only very high consumers of popular television images of medical worlds but also highly attuned to the potential relevance to their studies as measured by their awareness of the ethical and medical issues portrayed in the programs. However, this study was confined to one university in Australia and did not compare other student groups outside medicine. Many of our students are from culturally and linguistically diverse backgrounds (Table 1) and therefore may not reflect other medical student groups in Australia. Surveying medical students in other parts of Australia would provide valuable comparative data. Further research could also elucidate some of the contradictions that arose in our findings, such as the lead character of *House *nominated as both negative and positive role model. It may be that students who believe House is an appealing role model do so because of specific positive characteristics (for example, his intellect and commitment to solving medical mysteries) rather than - or despite - his negative traits (his contempt for patients). As Wicclair [10] points out, this determination to finding medical solutions may be part of the show's appeal. Given the problem-based learning format of medical education for these students, this may well be the appeal for them as well. Future research could address these areas in more depth.

## Conclusions

This study shows that medical students are high consumers of televisual representations of their chosen profession. This high level of engagement suggests that medical television programs may have some pedagogical value in the classroom. Students must be taught in a way that engages them and that does not ignore the potentially significant impact that cultural artefacts can have on their professional development and identity, while ensuring that their negotiation of popular culture gives them an authentic perception of the real medical world they are entering.

## Competing interests

The authors declare that they have no competing interests.

## Authors' contributions

RW conceptualised this study, collected and interpreted the data, and wrote and approved the final manuscript. IW analysed the data and wrote and approved the final manuscript.

## Pre-publication history

The pre-publication history for this paper can be accessed here:

http://www.biomedcentral.com/1472-6920/11/50/prepub
